# CARM1 S217 phosphorylation by CDK1 in late G2 phase facilitates mitotic entry

**DOI:** 10.1038/s41419-025-07533-z

**Published:** 2025-03-25

**Authors:** Yena Cho, Dae-Geun Song, Su-Nam Kim, Yong Kee Kim

**Affiliations:** 1 Muscle Physiome Research Center and Research Institute of Pharmaceutical Sciences, Sookmyung Women’s University, Seoul, 04310 Republic of Korea; 2 College of Pharmacy, Sookmyung Women’s University, Seoul, 04310 Republic of Korea; 3 Natural Products Research Institute, KIST Gangneung, Gangneung, 25451 Republic of Korea; 4 Natural Product Applied Science, KIST School, University of Science and Technology, Gangneung, 25451 Republic of Korea

**Keywords:** Mitosis, Kinases

## Abstract

The coactivator-associated arginine methyltransferase 1 (CARM1) functions as an epigenetic writer, however, its role in mitosis remains poorly understood. In this study, we identified CARM1 as a novel substrate of cyclin-dependent kinase 1 (CDK1) and revealed its novel function as a scaffold that regulates CDK1 stability. During interphase, CARM1 acts as an adaptor in the Cullin-1-mediated CDK1 degradation process, limiting nuclear levels of CDK1. In late G2 phase, the CDK1/Cyclin B1 complex translocates to the nucleus, where it phosphorylates the S217 residue of CARM1. This phosphorylation not only inhibits CARM1’s enzymatic activity but also facilitates its translocation to the cytoplasm, leading to the loss of its scaffolding function. Consequently, the CDK1/Cyclin B1 complex resides for longer in the nucleus and initiates mitosis. In addition, depletion or inhibition of CARM1 facilitates entry into mitosis, resulting in accelerated cell growth. Overall, our findings expand the cellular functions of CARM1 beyond its enzymatic activity.

## Introduction

Cyclin-dependent kinase 1 (CDK1) is an essential regulator of cell cycle progression, particularly during the G2/M transition [1, 2]. During late G2 phase, the accumulation of Cyclin B1 promotes the formation of the CDK1/Cyclin B1 complex, which is activated by a CDK1/Wee1/Cdc25 interlinked positive feedback loop [3–9]. The active CDK1/Cyclin B1 complex quickly moves into the nucleus *via* autophosphorylation of S126/128 within the cytoplasmic retention sequence (CRS) of Cyclin B1 [10–12]. Nuclear import of this complex is further enhanced by polo-like kinase 1 (PLK1)-mediated S133/147 phosphorylation within CRS. Subsequently, it phosphorylates various substrates, including histone H1 [13] and nuclear lamin [14, 15], triggering entry into mitosis [16]. Although the function of CDK1 in mitotic entry is well established, only a limited number of its substrates have been identified.

Coactivator-associated arginine methyltransferase 1 (CARM1) is a type I protein arginine methyltransferase (PRMT) that catalyzes the asymmetric dimethylation of arginine residues [17, 18]. It regulates multiple cellular processes including transcription [19, 20], RNA splicing [20, 21], and DNA repair [22, 23], affecting cell proliferation and differentiation. Recent studies have highlighted post-translational modifications (PTMs) of CARM1 and their implications [24]. Specifically, K471 ubiquitination of CARM1 by the S-phase kinase-associated protein 2 (Skp2)/Cullin-1 (CUL-1) ubiquitin ligase complex in the nucleus affects the transcription of autophagy-related genes [25]. The phosphorylation of S217 and S229 during mitosis impairs its binding to S-adenosyl-L-methionine and dimerization, respectively [26, 27]. It has been well established that CARM1 is phosphorylated at S229 by protein kinase C (PKC) [28]. However, the kinases responsible for S217 phosphorylation and the interplay between S217 and S229 phosphorylation are not well understood. Therefore, we aimed to identify the specific kinase responsible for CARM1 S217 phosphorylation and to clarify the cell cycle signaling pathways necessary for mitotic entry by investigating the interplay between S217 and S229 phosphorylation.

## Results

### CARM1 inhibition promotes rapid cell growth and mitotic entry

**Fig. 1:**
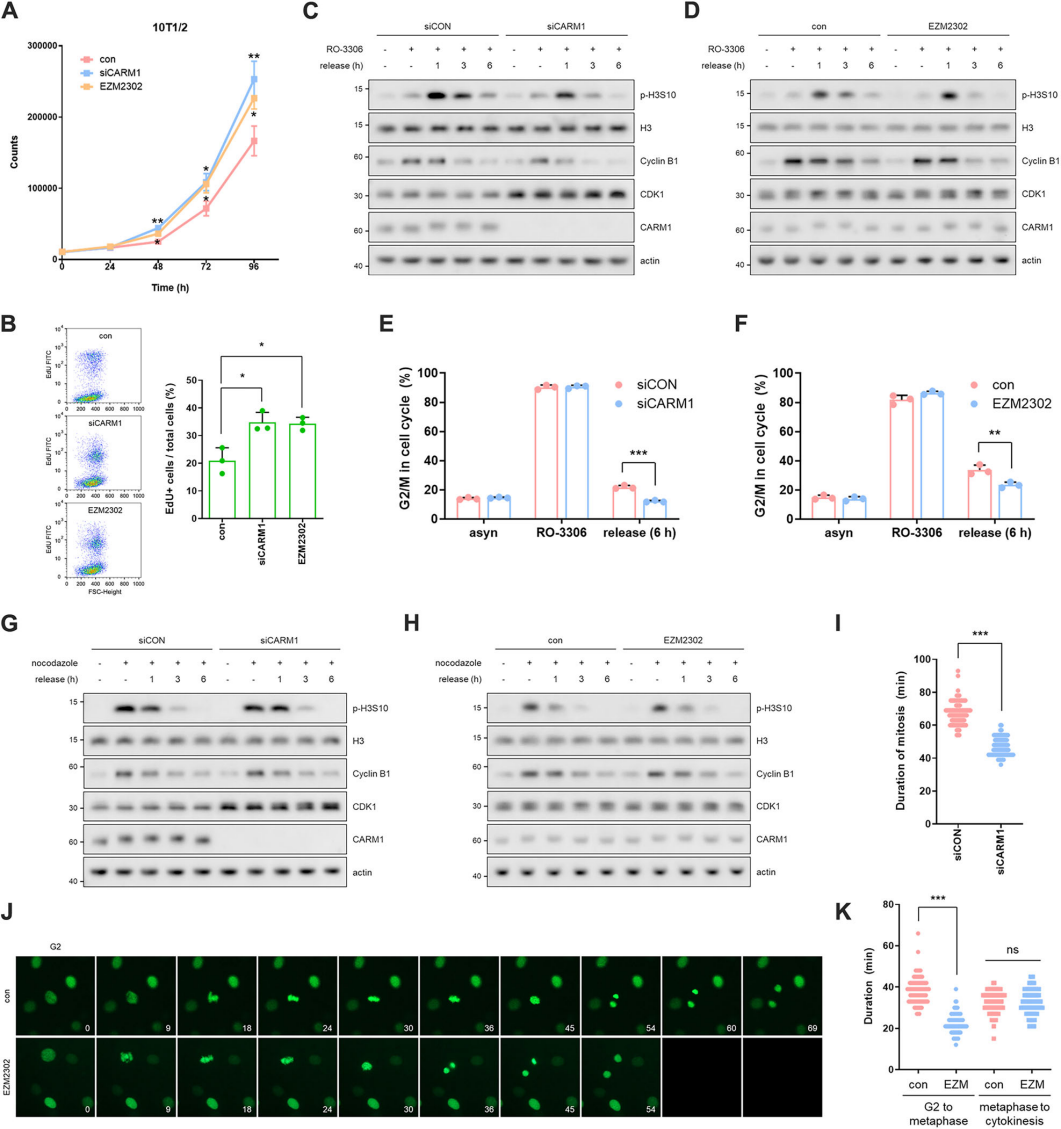
CARM1 inhibition promotes rapid cell growth and mitotic entry. **A** Growth curves of 10T1/2 cells treated with CARM1 siRNA or inhibitor for 72 h. Error bars indicate the SD of three independent experiments. **B** EdU incorporation assay using FACS in CARM1-depleted or -inhibited 10T1/2 cells. Data are presented as mean ± SD (*n* = 3). **C**, **D** Western blots of cell lysates from release experiment after G2 phase arrest in CARM1-depleted (**C**) or -inhibited (**D**) 10T1/2 cells. **E**, **F** Cell cycle analysis using FACS from release experiment after G2 phase arrest in CARM1-depleted (**E**) or -inhibited (**F**) 10T1/2 cells. Data are presented as mean ± SD (*n* = 3). **G**, **H** Western blots of cell lysates from release experiment after prometaphase arrest in CARM1-depleted (**G**) or -inhibited (**H**) 10T1/2 cells. **I** After transfection with CARM1 siRNA for 72 h, 10T1/2 cells stably expressing GFP-H2B were filmed for 5 h. The duration of mitosis was determined (*n* = 90). **J**, **K** After treatment with CARM1 inhibitor for 72 h, 10T1/2 cells stably expressing GFP-H2B were filmed for 5 h. Representative images are shown (**J**) and the duration of G2/M progression was determined (*n* = 90) (**K**).

We observed rapid growth in normal mouse embryonic fibroblasts (MEFs and 10T1/2 cells) when CARM1 was depleted or inhibited, as evidenced by cell counting (Figs. 1A and S1A) and the 5-ethynyl-2′-deoxyuridine (EdU) incorporation assay (Figs. 1B and S1B). To further characterize the effect of CARM1 on the cell growth rate, we analyzed cell cycle progression by measuring cyclin levels and flow cytometry-based DNA content. There were no differences in overall cell cycle profiles between asynchronous CARM1-wild type (WT) and -knockout (KO) MEF cells (Fig. S1C). Under double thymidine block (G1 arrest) and release settings, G1/S-specific Cyclin D3 levels also showed a similar pattern regardless of CARM1 expression, suggesting that CARM1 does not affect G1/S transition (Fig. S1D). However, in the late G2 block and release conditions with the CDK1 inhibitor RO-3306 [29], CARM1-depleted or -inhibited cells showed a faster decline in mitotic H3S10 phosphorylation (p-H3S10) and G2/M-specific Cyclin B1 levels (Figs. 1C, D and S1E), and a quicker recovery of the cell cycle profile (Figs. 1E, F and S1F–I). Interestingly, these findings were not observed under prometaphase arrest and release conditions with nocodazole (Fig. 1G, H), indicating that depletion or inhibition of CARM1 accelerated the G2/M transition. Furthermore, quantitative analysis of mitotic progression using time-lapse images of GFP-histone H2B expressing 10T1/2 cells showed shortened mitotic duration, particularly mitotic entry time, in CARM1-depleted or -inhibited cells (Fig. 1I–K; Videos S1, 2). Consequently, the disruption of CARM1 increases the rate of cell proliferation by promoting the transition from the G2 phase to mitosis.

### CDK1 phosphorylates CARM1 S217 and subsequently inhibits its activity during mitosis

**Fig. 2:**
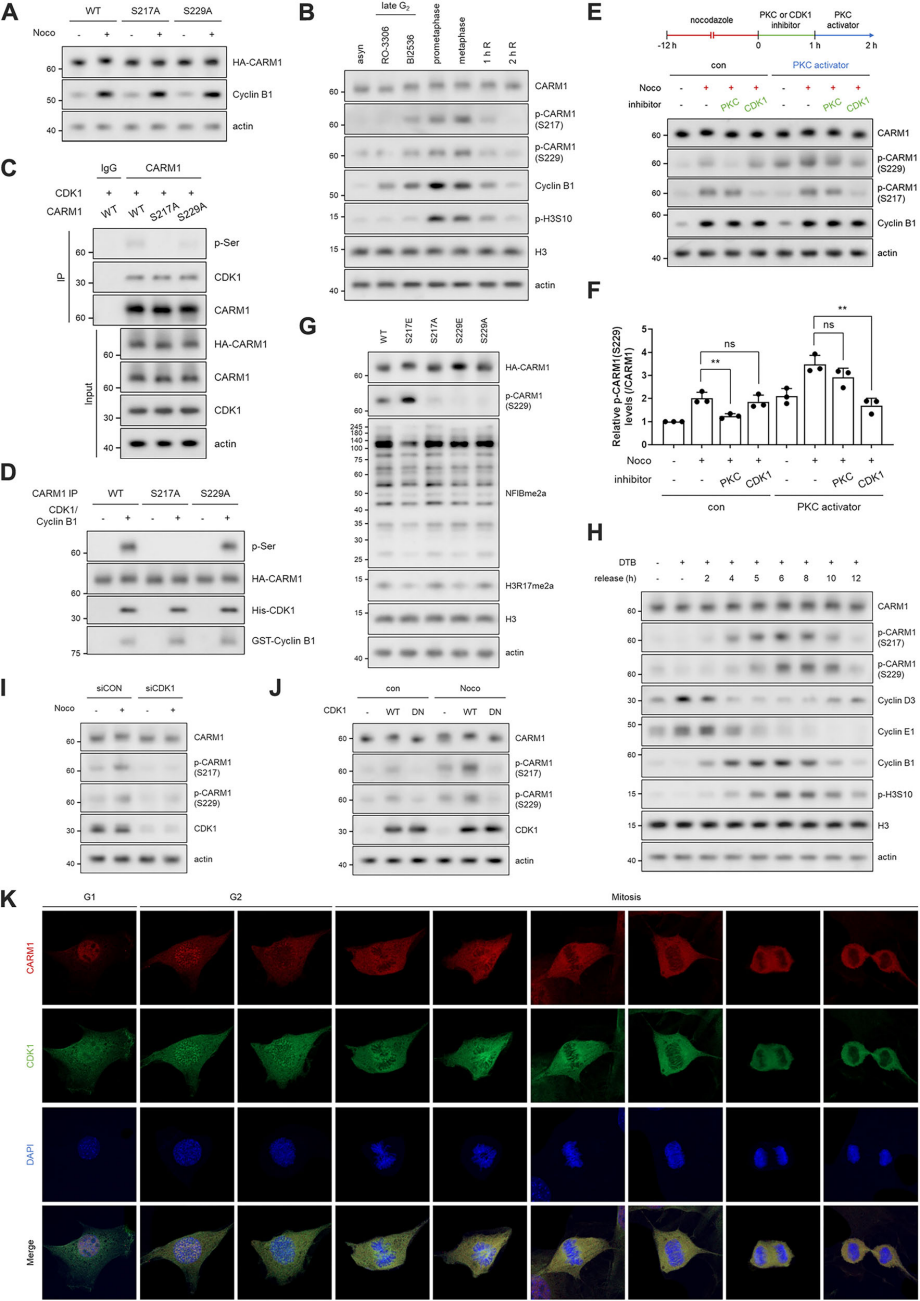
CDK1 phosphorylates CARM1 S217 and subsequently inhibits its activity during mitosis. **A** Western blots of cell lysates from nocodazole-treated cells after HA-CARM1 (WT, S217A, or S229A) transfection for 48 h. **B** Western blots of cell lysates from late G2, prometaphase, or metaphase-arrested cells. **C** IP using anti-CARM1 antibody in cells co-transfected with HA-CDK1 and HA-CARM1 (WT, S217A, or S229A) for 48 h. **D** In vitro kinase assay using recombinant CDK1/Cyclin B1 protein and beads carrying immunoprecipitated HA-CARM1 (WT, S217A, or S229A). **E**, **F** Western blots of cell lysates from cells under the indicated condition. After nocodazole pretreatment, calphostin C (0.5 µM, 1 h) or RO-3306 (10 µM, 1 h) was added, followed by PMA (100 nM, 1 h). Levels of p-CARM1 (S229) were normalized to total CARM1 levels and determined by three independent experiments. **G** Western blots of cell lysates from HA-CARM1 (WT, S217E, S217A, S229E, or S229A) overexpressing cells. NFIBme2a and H3R17me2a are substrates of CARM1. **H** Western blots of cell lysates from the release experiment after G1 phase arrest (double thymidine block, DTB). **I**, **J** Western blots of cell lysates from nocodazole-treated cells after transfection with CDK1 siRNA (**I**) or HA-CDK1 (WT or D146N) (**J**) for 48 h. **K** Confocal images of CARM1 (red), CDK1 (green), and DAPI (blue) in 10T1/2 cells.

Next, we focused on the band shift of CARM1, which was repeatedly observed during mitosis (Figs. 1C, D, G, H and S1D). Given previous reports that CARM1 is phosphorylated at S217 and S229 during mitosis [26, 27], we generated mutants in which S217 or S229 was substituted with E (phosphomimetic) or A (phosphodeficient). As expected, nocodazole treatment did not cause a CARM1 band shift in S217A or S229A mutant (Fig. 2A). To elucidate the role of S217 phosphorylation and identify the responsible kinase, we generated a CARM1 S217 phosphorylation-specific antibody. As shown in Fig. S2A, this antibody specifically recognized phosphorylated CARM1 at S217. We suggested that CDK1 was responsible for CARM1 phosphorylation during mitosis. This was because, under various conditions for G2/M synchronization—late G2 phase (using RO-3306 or BI2536, a PLK1 inhibitor), prometaphase (using nocodazole), or metaphase (using nocodazole release with MG132)—only RO-3306 treatment exclusively blocked the CARM1 band shift and phosphorylation (Fig. 2B). In support of this hypothesis, the cytoplasmic translocation of CARM1 *via* S217 phosphorylation [27] was blocked by RO-3306 treatment (Fig. S2B, C). In addition, our data provided clear evidence that CDK1 directly and exclusively phosphorylates CARM1 at S217; WT and catalytically active (CA) mutant of PKC, a known as kinase for S229 phosphorylation [28], had no effect on S217 phosphorylation (Fig. S2D), whereas CDK1 WT induced S217 phosphorylation (Fig. S2E). CDK1 phosphorylated CARM1 WT and S229A mutant, but not the S217A mutant, in both cellular systems (Fig. 2C) and in vitro CDK1/Cyclin B1 kinase assays (Fig. 2D). This notion was further supported by the fact that the peptide containing S217, but not the peptide containing S229, was phosphorylated by the CDK1/Cyclin B1 complex (Fig. S2F).

We then investigated the relationship between S217 and S229 phosphorylation because S229 phosphorylation decreased when S217 phosphorylation was inhibited (Fig. 2B, C). After synchronization at prometaphase with nocodazole, the cells were treated with specific inhibitors of PKC (calphostin C), CDK1 (RO-3306), or PLK1 (BI2536) for 1 h (Figs. 2E, F and S2G), followed by treatment with the PKC activator, phorbol 12-myristate-13-acetate (PMA) for an additional 1 h (Fig. 2E, F). Interestingly, RO-3306 blocked not only CARM1 S217 phosphorylation but also PKC-induced S229 phosphorylation (Fig. 2E, F), suggesting that CDK1-mediated S217 phosphorylation precedes PKC-mediated S229 phosphorylation. This was further supported by results showing that long treatment with RO-3306 (lane 6), similar to short treatment with calphostin C (lane 4), enabled CARM1 dimerization by blocking S229 phosphorylation (Fig. S2H). In addition, the S217E mutant, but not the S217A mutant, increased S229 phosphorylation (Fig. 2G). Moreover, analysis of CARM1 phosphorylation kinetics in cells synchronously released from G1 arrest revealed that S217 phosphorylation occurred first, followed by S229 phosphorylation during G2/M (Fig. 2H). Finally, we assessed CARM1 phosphorylation levels in either CDK1-knocked down or -overexpressing cells. As expected, CDK1 knockdown or dominant-negative mutant overexpression did not induce CARM1 phosphorylation, whereas overexpression of CDK1 WT increased CARM1 phosphorylation (Fig. 2I, J). Taken together, these results indicate that CDK1 is responsible for phosphorylating CARM1 at S217, which promotes its cytoplasmic translocation and subsequent phosphorylation at S229 during G2/M (Figs. 2K and S2I). This impairs CARM1’s enzymatic activity, as demonstrated by methylation levels determined using pan-CARM1 substrates antibody (NFIBme2a) and representative histone substrate antibody (H3R17me2a) (Figs. 2G and S2B).

### Mitotic progression requires an inactivation of CARM1 *via* S217 phosphorylation

**Fig. 3:**
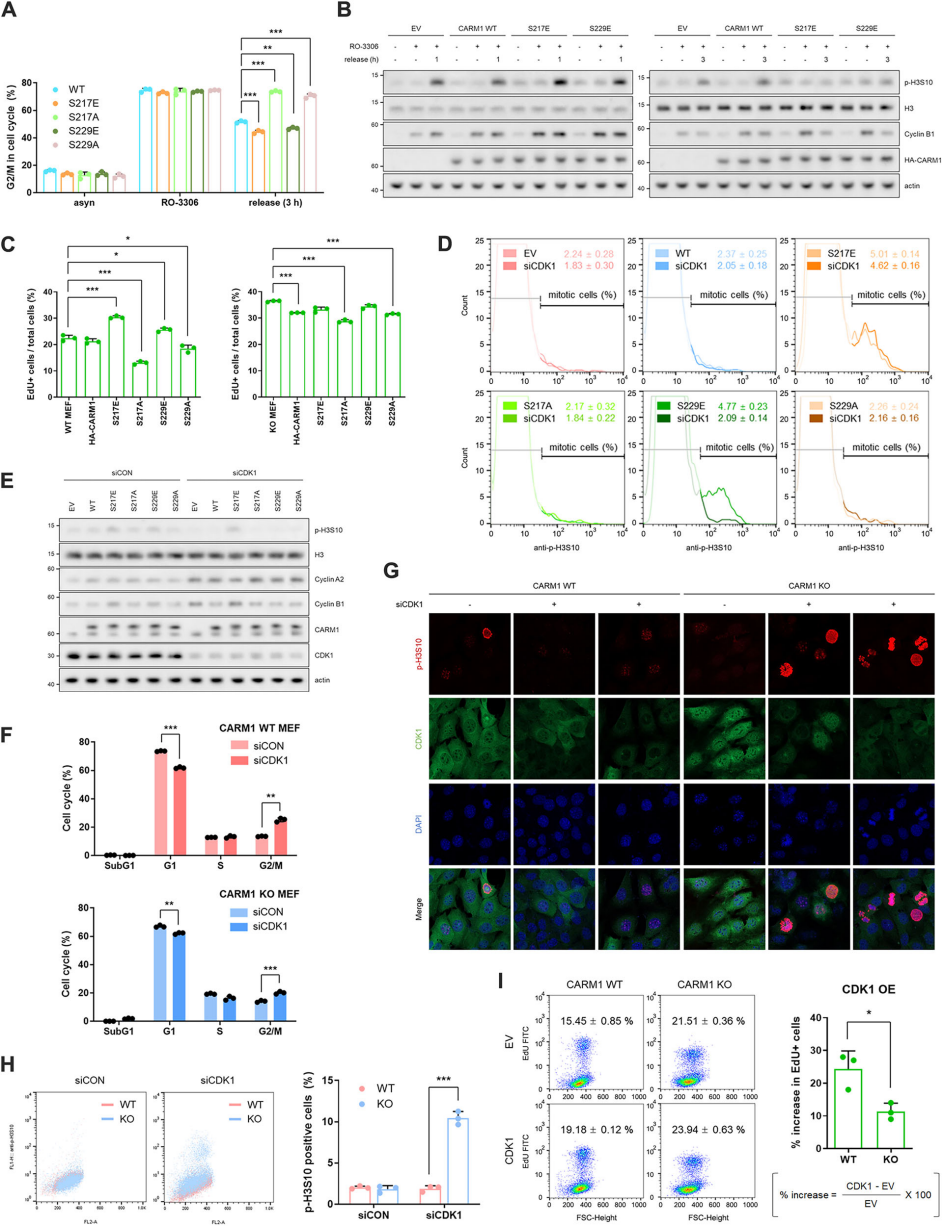
Mitotic progression requires an inactivation of CARM1 *via* S217 phosphorylation. **A** Cell cycle analysis using FACS from release experiment after G2 phase arrest in HA-CARM1 (WT, S217E, S217A, S229E, or S229A) overexpressing cells. Data are presented as mean ± SD (*n* = 3). **B** Western blots of cell lysates from the release experiment after G2 phase arrest in HA-CARM1 (WT, S217E, or S229E) overexpressing cells. **C** EdU incorporation assay using FACS in CARM-WT or -KO MEF cells transfected with HA-CARM1 (WT, S217E, S217A, S229E, or S229A) for 48 h. Data are presented as mean ± SD (*n* = 3). **D**, **E** Levels of p-H3S10 measured by FACS (**D**) or western blots (**E**) in cells co-transfected with HA-CARM1 (WT, S217E, S217A, S229E, or S229A) and CDK1 siRNA for 48 h. Data are presented as mean ± SD (*n* = 3). **F** Cell cycle analysis using FACS in CDK1-depleted CARM1-WT or -KO MEF cells. Data are presented as mean ± SD (*n* = 3). **G**, **H** Levels of p-H3S10 measured by confocal images (**G**) or FACS (**H**) in CDK1-depleted CARM1-WT or -KO MEF cells. Data are presented as mean ± SD (*n* = 3). **I** EdU incorporation assay using FACS in CARM-WT or -KO MEF cells transfected with HA-CDK1 for 48 h. Data are presented as mean ± SD (*n* = 3).

Our next question was whether CARM1 is inactivated for mitotic progression. From our earlier results showing that the duration of the G2/M transition was shortened in CARM1-depleted or -inhibited cells (Fig. 1C–F), we hypothesized that CARM1 activity is not necessary during the G2/M transition. Supporting this notion, CARM1 inactive mutants, S217E and S229E enabled cells to recover more quickly from RO-3306-induced G2 arrest (Figs. 3A, B and S3A). In contrast, S217A and S229A mutants failed to release G2-arrested cells (Figs. 3A and S3A, B). The introduction of either the S217E or S229E mutant into cells increased the cell growth rate, whereas the S217A or S229A mutant slowed cell growth (Figs. 3C and S3C). Notably, in CARM1-KO MEF cells, the inactive mutants (S217E and S229E) had no observable effect on cell proliferation (Figs. 3C and S3C), which appears to be attributable to the already enhanced growth caused by CARM1 depletion. To determine whether this finding resulted from CDK1-mediated CARM1 S217 phosphorylation, we co-transfected CARM1 WT or mutant with CDK1 siRNA. Except in S217E overexpressing cells, CDK1 knockdown induced G2 arrest, as evidenced by increased Cyclin A2 and B1 levels, without an increase in p-H3S10 (Figs. 3D, E and S3D). This suggests that CDK1 inactivates CARM1 *via* S217 phosphorylation, necessary for mitotic progression. We investigated the effect of CDK1 knockdown or overexpression in CARM1-WT and -KO MEF cells. CARM1 KO MEF cells bypassed CDK1 knockdown-induced G2 arrest (Figs. 3F–H and S3E, F) and attenuated the increase in cell growth rate induced by CDK1 overexpression (Figs. 3I and S3G). It is well established that CDK1 phosphorylates various substrates, including BUB1 mitotic checkpoint serine/threonine kinase [30], inner centromere protein [31], and Shugoshin 1 [32], to facilitate G2/M transition; all these CDK1-mediated phosphorylation cascades are important for mitotic progression and cell proliferation. Here, we identified CARM1 as a novel substrate critical for the G2/M transition. During the late G2 phase, CDK1 phosphorylates CARM1 at S217 priming S229 phosphorylation leading to its inactivation and promotion of mitotic entry.

### CARM1 functions as a scaffold for CUL-1-mediated CDK1 degradation

**Fig. 4:**
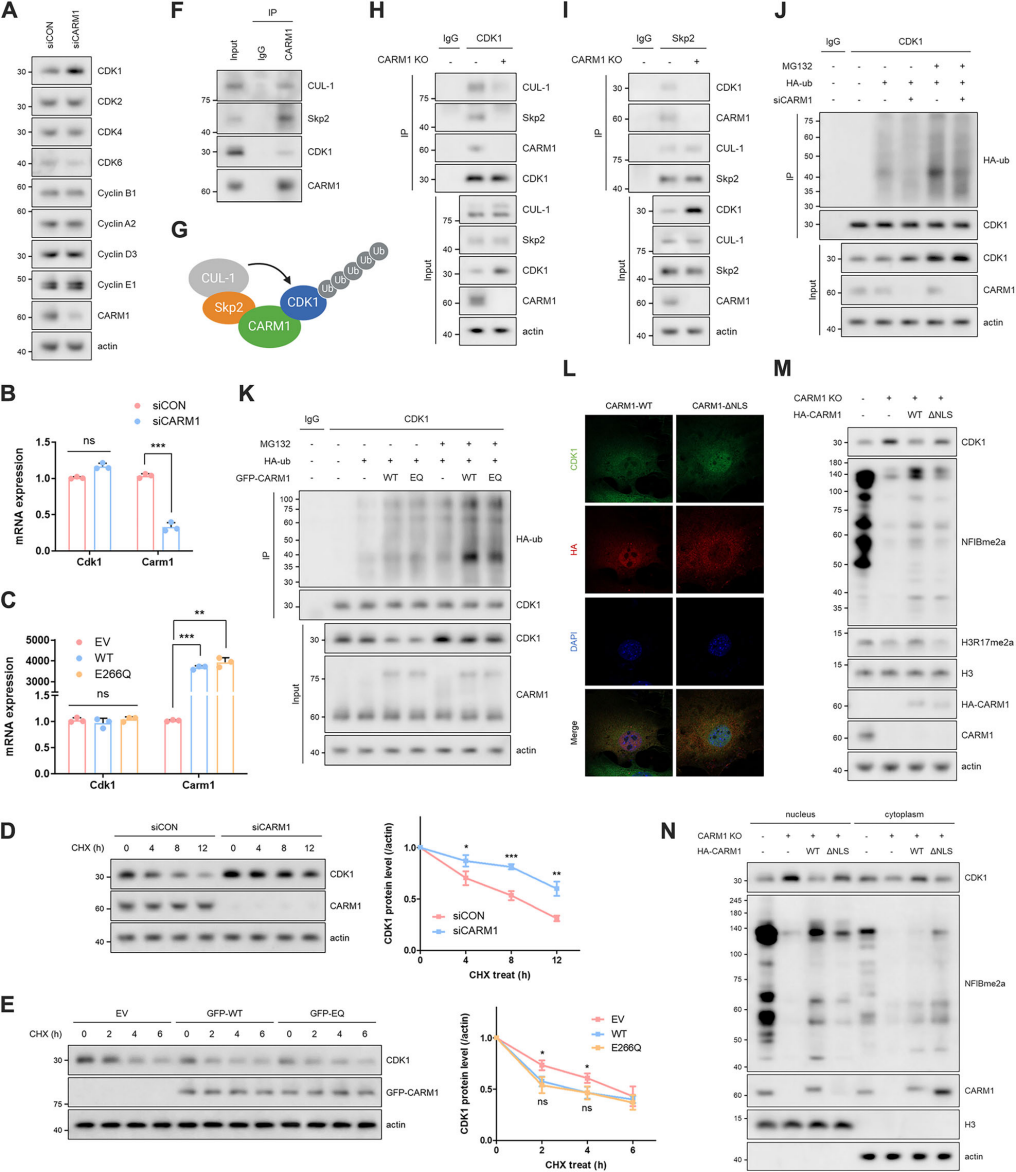
CARM1 functions as a scaffold for CUL-1-mediated CDK1 degradation. **A** Western blots of cell lysates from CARM1-depleted 10T1/2 cells. **B**, **C** mRNA levels of *CDK1* and *CARM1* in cells transfected with CARM1 siRNA for 72 h (**B**) or GFP-CARM1 (WT or E266Q) for 48 h (**C**). **D**, **E** Western blots of cell lysates from cells treated with cycloheximide (CHX, 50 μg/mL) for the indicated time after transfection with CARM1 siRNA for 72 h (**D**) or GFP-CARM1 (WT or E266Q) for 48 h (**E**). Error bars indicate the SD of three independent experiments. **F** IP of anti-CARM1 antibody in 10T1/2 cells. **G** Schematic of CUL-1-mediated CDK1 degradation depending on CARM1 as scaffold. **H**, **I** IP of anti-CDK1 (**H**) or anti-Skp2 (**I**) antibody in CARM1-WT or -KO MEF cells. **J**, **K** Ubiquitination assay in cells treated with MG132 (10 μM, 6 h) after co-transfection with either CARM1 siRNA (**J**) or GFP-CARM1 (WT or E266Q) (**K**) and HA-ub for 48 h. **L** Confocal images of CDK1 (green), HA (red), and DAPI (blue) in 10T1/2 cells overexpressing HA-CARM1 (WT or ΔNLS). **M**, **N** Western blots of total lysates (**M**) and nuclear/cytoplasmic fractions (**N**) from CARM1-KO MEF cells transfected with HA-CARM1 (WT or ΔNLS) for 48 h.

Close examination of Figs. S1D and S4A revealed an interesting observation: CDK1 levels were dramatically elevated, particularly in the nucleus (Fig. 3G), in CARM1-KO MEF cells. To further validate this observation, we transiently knocked down CARM1 and observed a marked increase in CDK1 protein levels; other CDKs and cyclins remained relatively consistent (Fig. 4A). While CARM1 depletion tended to increase *CDK1* mRNA levels, this effect was not statistically significant. Similarly, CARM1 overexpression did not influence *CDK1* mRNA levels (Figs. 4B, C and S4B); however, the regulation of CDK1 protein stability was clearly influenced by CARM1 depletion or overexpression (Figs. 4D, E and S4C). Interestingly, this regulation was unaffected by treatment with a CARM1 inhibitor (EZM2302) (Fig. S4D) or overexpression of the enzyme-dead mutant (E266Q) (Figs. 4E and S4E), suggesting that CARM1 may act as a scaffold for CDK1 degradation. Since CARM1 is ubiquitinated and degraded by the Skp2/CUL-1 complex in the nucleus [25], we examined whether CDK1 belongs to this complex. The co-immunoprecipitation assay revealed that CDK1 interacts with the CARM1/Skp2/CUL-1 complex (Fig. 4F), which was lost in CARM1-KO MEF cells (Fig. 4H, I), highlighting that CARM1 bridges CDK1 degradation by linking CDK1 to the Skp2/CUL-1 complex (Fig. 4G). Indeed, CARM1 knockdown reduced CDK1 ubiquitination (Fig. 4J), whereas CARM1 overexpression increased CDK1 ubiquitination regardless of its enzymatic activity (Fig. 4K). Furthermore, the introduction of CARM1 Δ348-381 (nuclear localization sequence, NLS) into CARM1 KO MEF cells did not affect CDK1 levels in the nucleus, unlike CARM1 WT (Fig. 4L–N), supporting the hypothesis that nuclear CARM1/Skp2/CUL-1 complex mediates CDK1 degradation. In addition, we confirmed whether cytoplasm-enriched S217E mutant (Fig. S2B) has less influence on CDK1 degradation compared to WT. The S217E mutant failed to serve as a bridge between the Skp2/CUL-1 complex, resulting in decreased CDK1 ubiquitination (Fig. S4F) and consequently, increased CDK1 levels (Fig. S4G). Therefore, we concluded that depletion or cytoplasmic translocation of CARM1 promotes mitotic entry by inhibiting CUL-1-mediated CDK1 degradation in the nucleus.

## Discussion

The cell cycle progression is tightly controlled by CDK/cyclin complexes that coordinate the orderly transition between each stage. The precise regulation of these complexes ensures the fidelity of cell division, preventing genomic instability and maintaining cellular homeostasis [33]. CDK2, 4 and 6 regulate progression from G1 to S phase, while CDK1 primarily drives entry into mitosis [34]. CDK activities are finely tuned by the timely expression of cyclin proteins, which fluctuate throughout the cell cycle *via* the ubiquitin-proteasome pathway [35, 36]. While it is widely accepted that CDK levels remain relatively consistent throughout the cell cycle, the mechanisms regulating these levels are not yet fully elucidated. Recent reports indicate that CDK1 levels are influenced by deubiquitinases such as YOD1 [37] and ubiquitin specific peptidase 7 [38]. Specifically, YOD1 binds directly to CDK1, leading to de-polyubiquitylation and upregulation of CDK1 expression [37]. We here highlighted a novel function of CARM1 in regulating intracellular CDK1 levels. Although CARM1 methylates arginine residues on substrate proteins, its enzymatic activity is not necessary for determining CDK1 levels. Instead, it functions solely as an adapter, bridging CDK1 to the Skp2/CUL-1 ubiquitin ligase complex. These findings suggest a scaffolding role of CARM1 beyond its enzymatic activity, which is supported by another report: CARM1 interacts with poly(ADP-ribose) polymerase 1 at replication forks and reduces fork speed, independently of its methyltransferase activity [39]. Considering these points, it is necessary to thoroughly investigate not only its enzymatic activity but also its protein itself when targeting CARM1 in various disease models.

PTMs of CARM1 are increasingly recognized for their biological significance. Among these, phosphorylation at S217, S229, S448, or S595 has been emerged as crucial regulator of its activity or subcellular localization [40–42]. Phosphorylation at S448 by PKA facilitates CARM1’s interaction with estrogen receptor α [40], while p38γ MAPK-mediated phosphorylation at S595 influences its subcellular localization [41, 42]. Phosphorylation at S217 and S229 occurs during mitosis, with S229 phosphorylation catalyzed by PKC [26–28]. In this study, we identified CDK1 as the kinase responsible for phosphorylating CARM1 at S217, which precedes phosphorylation at S229, with both events peaking during mitosis and sharply decreasing as the cell cycle transition from mitosis to G1 phase. These phosphorylation abolishes its methyltransferase activity, indicating that CARM1 activity is inhibited during mitosis. However, it is not fully understood why CARM1’s enzymatic activity needs to be inhibited. Our findings—demonstrating that EZM2302 treatment induces rapid cell growth (see Fig. 1) and that the CARM1 S217E mutation or KO bypasses G2 arrest caused by CDK1 depletion (see Fig. 3)—suggest that CARM1 regulates additional mitotic processes beyond stabilizing CDK1. Given CARM1’s well-defined role as an epigenetic writer, it is possible that an intricate interplay exists between histone marks, including H3R17me2a, in regulating chromosome condensation. Indeed, our data reveal that CARM1 inactive mutants (S217E and S229E) led to decreased H3R17me2a levels (Fig. 2G) and increased p-H3S10 levels (Fig. 3E). This approach will contribute to a comprehensive understanding of how chromosome condensation is initiated during mitosis, which is part of our ongoing study.

Our study elucidated the role of CARM1 in cell cycle regulation. We observed distinct differences in the duration of mitosis between CARM1 WT and KO cells, and focused on the effect of CARM1 in G2/M transition. However, in CARM1-depleted cells, the decrease in EdU intensity (Fig. 1B) and CDK6 levels (Fig. 4A) also suggests that CARM1 may influence other phases of the cell cycle. Moreover, a recent report indicating that CARM1 slows replication fork speed [39] implies that loss of CARM1 may shorten S phase. Although the current study did not address these aspects, further investigations are warranted to fully understand how CARM1 regulates cell cycle progression and growth.

## Conclusion

**Fig. 5:**
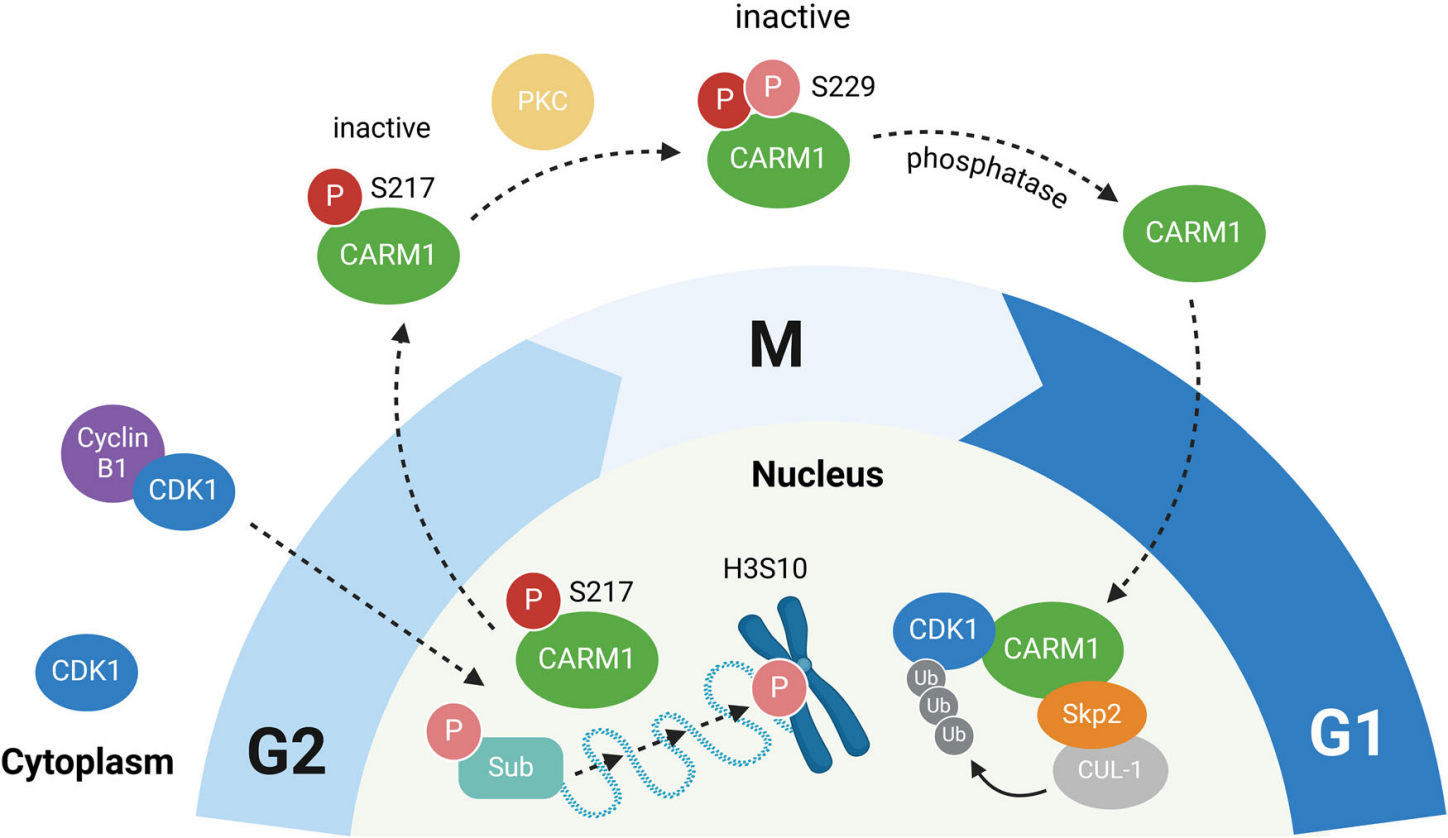
Crosstalk between CARM1 and CDK1 during mitosis. In the late G2 phase, the CDK1/Cyclin B1 complex enters the nucleus and phosphorylates CARM1 at position S217. This phosphorylation inhibits CARM1’s enzymatic activity and promotes its translocation into the cytoplasm. Because CARM1 acts as an adapter in Cullin-1-mediated CDK1 degradation in the nucleus, its cytoplasmic localization causes a loss of scaffolding function and stabilization of CDK1. Consequently, the CDK1/Cyclin B1 complex remained in the nucleus for a longer time, thereby initiating mitosis.

In summary, we described the crosstalk between CARM1 and CDK1 during mitosis (Fig. 5). In the late G2 phase, CDK1 phosphorylates the S217 residue of CARM1, inhibiting its enzymatic activity and promoting its translocation to the cytoplasm. Since CARM1 functions as an adapter regulating CDK1 stability within the nucleus, its cytoplasmic translocation during the late G2 phase allows CDK1 to remain stable or longer in the nucleus, facilitating mitotic entry. Taken together, we unraveled the intricately regulated mechanism of mitotic entry through CARM1’s scaffolding function, highlighting that CARM1’s cellular roles extend beyond its enzymatic activity.

## Materials and methods

### Materials

Key resource materials, including antibodies, chemicals, oligonucleotides, plasmids, proteins, and cell lines, are listed in Supplementary Information.

### Cell culture

10T1/2, MEF, and HEK293T cells were cultured in Dulbecco’s modified Eagle’s medium (HyClone, Logan, UT, USA) supplemented with 10% fetal bovine serum (HyClone) and 100 U/mL penicillin/streptomycin (HyClone). All cells were maintained at 37 °C in a humidified incubator with 5% CO_2_.

### Cell counting

Cells were seeded into 12-well plates at a density of 10,000 cells/well. After trypsinization, the cells were washed with phosphate-buffered saline (PBS) and counted using a Z2 Coulter Counter (Beckman Coulter, Brea, CA, USA). The diameters of the counted cells were in the range of 10–30 µm.

### EdU incorporation assay

Cells were seeded into 6-well plates and pulse-labeled with 20 µM EdU for 2 h. After trypsinization, the cells were washed with 3% bovine serum albumin in PBS and fixed with 4% formaldehyde. After 15 min of incubation in the dark, the cells were washed and permeabilized. An EdU reaction mixture containing CuSO_4_, iFluor 488 azide, and sodium ascorbate was prepared and added to cells. The samples were incubated for 30 min in the dark, washed, and transferred to new tubes. Data were measured using the FACSCalibur system (BD Biosciences, Franklin Lakes, NJ, USA) and analyzed using FlowJo software.

### Cell synchronization and flow cytometry analysis

To synchronize the G1 phase, the cells were seeded at 30% confluence and maintained in media containing 2 mM thymidine for 18 h. The cells were washed with PBS and placed in the fresh media for 9 h. Thereafter, the cells were incubated in the media supplemented with 2 mM thymidine for 15 h. To synchronize cells in the G2/M phase, cells were treated with RO-3306 (10 μM, 24 h), BI2536 (100 nM, 24 h), or nocodazole (100 ng/mL, 12 h). For metaphase arrest, cells were released in fresh media containing 20 μM MG132 for 2 h after nocodazole treatment.

To analyze the cell cycle, the cells were harvested using trypsin and fixed with 70% ethanol for 1 h on ice. The cells were washed with PBS and incubated with 0.2 mg/mL RNase A for 1 h at room temperature. An anti-p-H3S10 antibody (Alexa Fluor 488 conjugate) was used to distinguish mitotic cells. After harvesting, the cells were fixed with 4% formaldehyde for 15 min at room temperature and permeabilized with 90% methanol for 10 min on ice. The cells were then washed with PBS and incubated with a diluted primary antibody (1:50) for 1 h in the dark. Thereafter, the cells were stained with 10 μg/mL propidium iodide. Data were generated using a FACSCalibur system and analyzed using FlowJo software.

### Immunofluorescence and live cell imaging

Cells plated on coverslips were fixed with 4% paraformaldehyde and permeabilized with 0.5% Triton X-100 for 15 min at room temperature. After washing with PBS, cells were incubated with a diluted primary antibody (1:500) overnight at 4 °C, followed by incubation with a secondary antibody conjugated to FITC and/or Alexa Fluor 594. Staining was visualized using a Zeiss LSM 710 Confocal Microscope (Carl Zeiss, Oberkochen, Germany) and images were analyzed using ZEN.

For time-lapse microscopy, 10T1/2 cells stably expressing GFP-H2B were cultured in Leibovitz’s L-15 medium (Invitrogen, Waltham, MA, USA) supplemented with 10% fetal bovine serum and 2 mM L-glutamine (Invitrogen). The cells were placed in a sealed growth chamber heated to 37 °C and observed using a Zeiss Axiovert 200 M microscope (Carl Zeiss). Images were acquired every 3 min for 5 h using AxioVision 4.8.2 (Carl Zeiss).

### Immunoblotting and immunoprecipitation

As described previously [43], cells were lysed using RIPA lysis buffer (50 mM Tris-HCl [pH 8], 150 mM NaCl, 0.5% sodium deoxycholate, 0.1% sodium dodecyl sulfate, and 1% Triton X-100) supplemented with a 1× protease and phosphatase inhibitor cocktail. Sonication was used to lyse cells, and the lysates were centrifuged at 16,000 × *g* for 10 min at 4 °C. The protein concentration of the lysates was quantified using Bradford assay (Bio-Rad, Hercules, CA, USA).

After lysate centrifugation and protein quantification, appropriate antibody was added to the samples for immunoprecipitation. The mixture was incubated overnight at 4 °C on a rotator. Antibody-protein complexes were captured using protein A/G Sepharose beads by placing the mixture on a rotator for 2 h at 4 °C. After washing twice with lysis buffer, the complexes were eluted and separated using sodium dodecyl sulfate-polyacrylamide gel electrophoresis (SDS-PAGE). The separated proteins were transferred onto a polyvinylidene fluoride membrane (Millipore, Billerica, MA, USA) and blocked with 0.1% Tween 20/Tris-buffered saline (TBS-T) containing 5% skim milk for at least 1 h at room temperature. Subsequently, the membrane was incubated with a primary antibody overnight at 4 °C. After washing three times with TBS-T, the membrane was incubated with a horseradish peroxidase-conjugated secondary antibody for 1 h at room temperature. The signal was detected using an ECL western blotting substrate (Advansta, Menlo Park, CA, USA).

### In vitro kinase assay

In vitro kinase assay used recombinant CDK1/Cyclin B1 protein, immunoprecipitated HA-CARM1 from HEK293T cells or CARM1(212-222 or 224-234) peptide (YAVEASTMAQH or EVLVKSNNLTD) (Anygen, Gwangju, Korea), and 1 mM ATP in kinase buffer (25 mM Tris-HCl [pH 7.5], 150 mM NaCl, 2 mM DTT, and 0.1 mM Na_3_VO_4_, 10 mM MgCl_2_) supplemented with 1× protease and phosphatase inhibitor cocktail. The samples were incubated at 37 °C for 1 h, and the proteins were separated by SDS-PAGE.

### Subcellular fractionation

Cells were scraped from the culture dish and washed with PBS. After collecting the cell pellet, the cells were gently resuspended in a hypotonic lysis buffer (10 mM HEPES [pH 7.5], 10 mM MgCl_2_, and 20 mM KCl) and incubated for 10 min on ice. Thereafter, 0.5% NP-40 was added and homogenates were centrifuged at 700 × *g* for 10 min at 4 °C. The supernatant containing the cytoplasmic fraction was then transferred to a new tube. The pellet containing the nuclear fraction was resuspended with RIPA lysis buffer and sonicated. After centrifugation at 16,000 × *g* for 10 min at 4 °C, the supernatant was transferred to a new tube.

### Mutagenesis

As described previously, a GFP-CARM1 mutant (E266Q) was generated using the Muta-Direct Site-Directed Mutagenesis Kit, following the manufacturer’s protocol [44]. The other HA-CARM1 mutants were produced by BIONICS (Seoul, Korea).

### Quantitative real-time PCR

Total RNA was extracted using TRIzol reagent; chloroform was added to the cell lysate. The mixture was then vortexed and incubated on ice for 10 min. After centrifugation at 16,000 × *g* for 15 min at 4 °C, the colorless aqueous phase containing RNA was transferred to a new tube and isopropanol was added. After incubation for 10 min at room temperature, the samples were centrifuged at 16,000 × *g* for 10 min and the pellet was washed with 70% ethanol. The dried RNA pellet was resuspended in nuclease-free water. The cDNA was synthesized using the SensiFAST cDNA Synthesis Kit. Further, mRNA expression was analyzed using the QuantStudio 3 Real-Time PCR System (Applied Biosystems, Foster City, CA, USA), SensiFAST SYBR No-ROX Kit, and the ΔΔCT method. The reaction parameters were as follows: cDNA synthesis at 40 °C for 60 min, transcriptase inactivation at 95 °C for 5 min, and PCR cycling at 95 °C for 10 s, 58 °C for 20 s, and 72 °C for 20 s (40 cycles).

### Statistical analysis

All statistical analyses were performed using the Prism software (GraphPad). Data are representative of independent experiments and have been presented as mean ± standard deviation (*n* = 3). Data from two groups were compared using unpaired *t*-test for independent samples. Statistical significance was set at *p* < 0.05. *p < 0.05, **p < 0.01, and ***p < 0.001.

## Supplementary information

Supplementary information

Supplemental material - WB raw data

Video S1

Video S2

## Data Availability

Supplementary information is available with this paper. All data reported in this paper will be shared by the corresponding author upon reasonable request. Any additional information required to reanalyze the data reported in this paper is available from the corresponding author upon reasonable request.
