# Supplementary material for: CARM1 S217 phosphorylation by CDK1 in late G2 phase facilitates mitotic entry
Source: Cell Death Dis. 2025 Mar 25;16(1):202. doi: 10.1038/s41419-025-07533-z (PMC11937338; doi:10.1038/s41419-025-07533-z)
Supplement: Supplementary file 1 — Supplementary information [file 41419_2025_7533_MOESM1_ESM.docx]

**[Supplementary Information]**

**CARM1 S217 phosphorylation by CDK1 in late G2 phase facilitates mitotic entry**

Yena Cho^1,2^, Dae-Geun Song^3,4^, Su-Nam Kim^3,4^, and Yong Kee Kim^1,2,*^

^1^ Muscle Physiome Research Center and Research Institute of Pharmaceutical Sciences, Sookmyung Women’s University, Seoul 04310, Republic of Korea

^2^ College of Pharmacy, Sookmyung Women’s University, Seoul 04310, Republic of Korea

^3^ Natural Products Research Institute, KIST Gangneung, Gangneung 25451, Republic of Korea

^4^ Division of Bio-Medical Science and Technology, University of Science and Technology KIST School, Seoul 02792, Republic of Korea

***Correspondence:**

Yong Kee Kim, Ph.D., E-mail: [yksnbk@sookmyung.ac.kr](mailto:yksnbk@sookmyung.ac.kr), Tel: +82-2-2077-7688, Fax: +82-2-710-9871

**Supplementary Video**

**Video S1: A representative time-lapse video of mitotic division of 10T1/2 cells stably expressing GFP-H2B.** Left side: siCON, right side: siCARM1.

**Video S2: A representative time-lapse video of mitotic division of 10T1/2 cells stably expressing GFP-H2B.** Left side: control, right side: EZM2302.

**Table I. Key resource materials**

| **Reagent or resource** | **Source** | **Identifier** |
| --- | --- | --- |
| **Antibodies** | | |
| Actin | Santa Cruz Biotechnology | sc-47778 |
| CARM1 | Bethyl Laboratories | A300-421A |
| p-CARM1 (S217) | Abfrontier | This paper |
| p-CARM1 (S229) | Cusabio | CSB-PA447549 |
| CDK1 | Santa Cruz Biotechnology | sc-54 |
| CDK2 | Cell Signaling Technology | #2546 |
| CDK4 | Cell Signaling Technology | #2906 |
| CDK6 | Cell Signaling Technology | #3136 |
| CUL-1 | Santa Cruz Biotechnology | sc-17775 |
| Cyclin A2 | Cell Signaling Technology | #81754 |
| Cyclin B1 | Cell Signaling Technology | #12231 |
| Cyclin D3 | Cell Signaling Technology | #2936 |
| Cyclin E1 | Santa Cruz Biotechnology | sc-377100 |
| GFP | Santa Cruz Biotechnology | sc-9996 |
| GST | Santa Cruz Biotechnology | sc-138 |
| HA | Cell Signaling Technology | #3724 |
| His | Santa Cruz Biotechnology | sc-803 |
| Histone H3 | Cell Signaling Technology | #9715 |
| H3R17me2a | Abcam | ab8284 |
| p-H3S10 | Cell Signaling Technology | #9701 |
| Alexa Fluor 488-conjugated p-H3S10 | Cell Signaling Technology | #3465 |
| NFIBme2a | Dr. Mark T Bedford (MD Anderson Cancer Center, USA) | N/A |
| p-Ser | Santa Cruz Biotechnology | sc-81514 |
| Skp2 | Santa Cruz Biotechnology | sc-7164 |
| Ub | Santa Cruz Biotechnology | sc-8017 |
| Alexa Fluor-conjugated secondary antibodies | Bethyl Laboratories | A90-116D4  A90-138D2  A120-101D4  A120-101F |
| Horseradish peroxidase-conjugated secondary antibodies | Jackson ImmunoResearch Laboratories | 111-035-003  115-035-003 |
| **Chemicals and reagents** | | |
| 4′,6-diamidino-2-phenylindole (DAPI) | Thermo Fisher Scientific | D1306 |
| BI2536 | MedChemExpress | HY-50698 |
| Calphostin C | Sigma-Aldrich | C6303 |
| Cycloheximide | Sigma-Aldrich | C4859 |
| EZM2302 | MedChemExpress | HY-111109 |
| MG132 | Enzo Life Sciences | BML-PI102 |
| Nocodazole | Sigma-Aldrich | 487928 |
| Phorbol 12-myristate 13-acetate (PMA) | Sigma-Aldrich | P8139 |
| Propidium iodide | Sigma-Aldrich | P4170 |
| RNase A | Thermo Fisher Scientific | R1253 |
| RO-3306 | Sigma-Aldrich | SML0569 |
| Thymidine | Sigma-Aldrich | T1895 |
| 1,4-dithiothreitol (DTT) | Roche | 3483-12-3 |
| cOmplete | Roche | 11836153001 |
| PhosSTOP | Roche | 4906837001 |
| Protein A/G Sepharose beads | Santa Cruz Biotechnology | sc-2003 |
| TransIT-2020 | Mirus Bio | MIR 5400 |
| TransIT-X2 | Mirus Bio | MIR 6000 |
| **Plasmids** | | |
| GFP-CARM1 | Dr. Mark T Bedford (MD Anderson Cancer Center, USA) | N/A |
| GFP-CARM1 (E266Q) | This paper | N/A |
| HA-CARM1 | Addgene | #81118 |
| HA-CARM1 (ΔNLS) | This paper | N/A |
| HA-CARM1 (S217A) | This paper | N/A |
| HA-CARM1 (S217E) | This paper | N/A |
| HA-CARM1 (S229A) | This paper | N/A |
| HA-CARM1 (S229E) | This paper | N/A |
| HA-CDK1 | Addgene | #1888 |
| HA-CDK1 (D146N) | Addgene | #1889 |
| HA-PKCα | Addgene | #21232 |
| HA-PKCα (CA) | Addgene | #21234 |
| HA-PKCα (DN) | Addgene | #21235 |
| HA-ub | Addgene | #18712 |
| **Oligonucleotides** |  |  |
| *Actb*  F: ACCTTCTACAATGAGCTGCG  R: CTGGATGGCTACGTACATGG | This paper | N/A |
| *Carm1*  F: GTTTTCAAGTGCTCGGTGTC  R: CGACAGGTTTTCAGGATGTTG | This paper | N/A |
| *Cdk1*  F: TGCAGGACTACAAGAACACC  R: GCCATTTTGCCAGAGATTCG | This paper | N/A |
| **Recombinant protein** |  |  |
| CDK1/Cyclin B1, active | Sigma-Aldrich | 14-450 |
| **Bacterial and virus strains** |  |  |
| Chemically competent *E. coli* DH5α | Enzynomics | CP011 |
| **Critical commercial assays** | | |
| EdU Proliferation Kit (iFluor 488) | Abcam | ab219801 |
| Fast Ion Plasmid Midi Kit | RBC Bioscience | YP125 |
| Intracellular Flow Cytometry Kit (Methanol) | Cell Signaling Technology | #13593 |
| Muta-Direct Site Directed Mutagenesis Kit | iNtRON Biotechnology | #15071 |
| SensiFAST cDNA Synthesis Kit | Bioline | BIO-65053 |
| SensiFAST SYBR No-ROX Kit | Bioline | BIO-98005 |
| TRIsure | Bioline | BIO-38033 |
| **Experimental models: Cell lines** | | |
| 10T1/2 | ATCC | CCL-226 |
| GFP-H2B stable 10T1/2 | This paper | N/A |
| CARM1 WT MEF | Dr. Mark T Bedford (MD Anderson Cancer Center, USA) | N/A |
| CARM1 KO MEF | Dr. Mark T Bedford (MD Anderson Cancer Center, USA) | N/A |
| HEK293T | ATCC | CRL-3216 |
| **Software and algorithms** | | |
| FlowJo | https://www.flowjo.com/solutions/flowjo/ | N/A |
| Image Studio Lite Version 5.0 | https://www.licor.com/bio/image-studio/ | N/A |
| Prism | https://www.graphpad.com/scientific-software/prism/ | N/A |

**
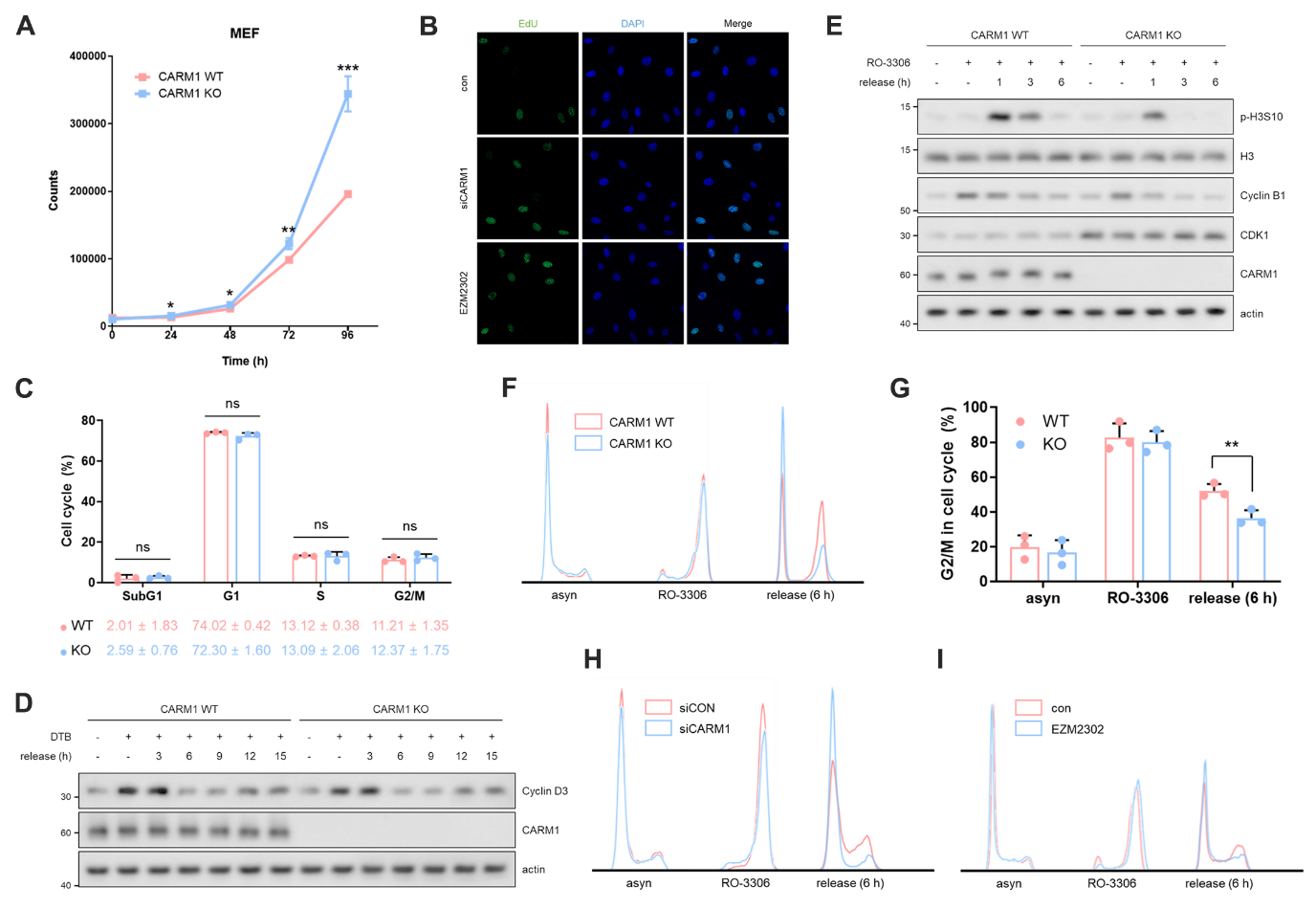
**

**Fig. S1. CARM1 inhibition promotes rapid cell growth and mitotic entry.**

(**A**) Growth curves of CARM1-WT and -KO MEF cells. The error bars indicate the SD of three independent experiments.

(**B**) EdU incorporation assay using confocal microscope in CARM1-depleted or -inhibited 10T1/2 cells.

(**C**) Cell cycle profiles in asynchronous CARM1-WT and -KO MEF cells.

(**D** and **E**) Western blots of cell lysates from the release experiment after G1 phase (D) or G2 phase (E) arrest in CARM1-WT or -KO MEF cells.

(**F** and **G**) Cell cycle analysis using FACS from release experiment after G2 phase arrest in CARM1-WT or -KO MEF cells. Data are presented as mean ± SD (n = 3).

(**H** and **I**) Cell cycle analysis using FACS from release experiment after G2 phase arrest in CARM1-depleted (G) or -inhibited (H) 10T1/2 cells.


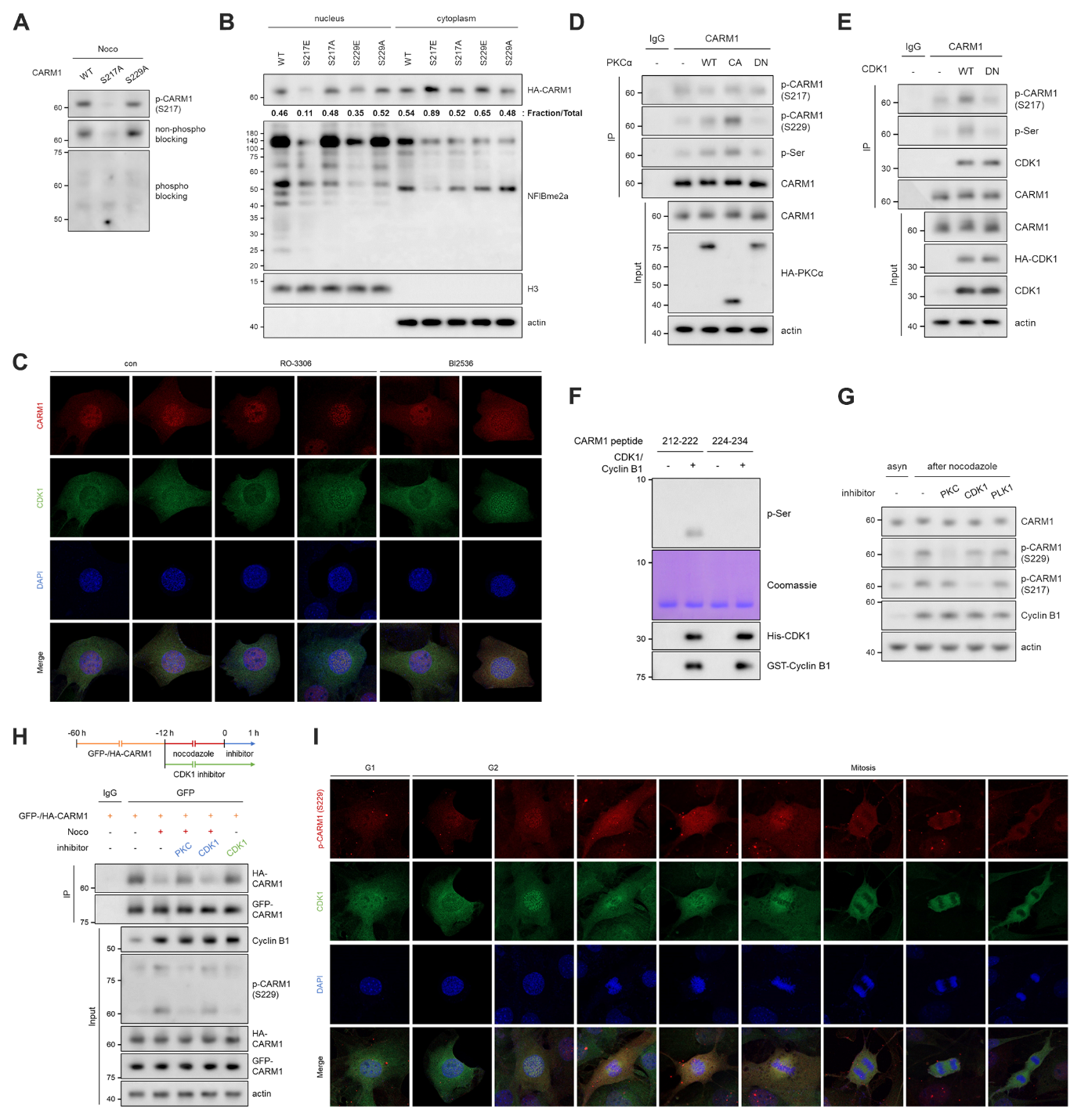


**Fig. S2. CDK1 phosphorylates CARM1 S217 and subsequently inhibits its activity during mitosis.**

(**A**) Western blots of cell lysates from HA-CARM1- (WT, S217A, or S229A) overexpressing cells treated with nocodazole. Blocking test using non-phospho or phospho peptide was performed to validate anti-p-CARM1 (S217) antibody.

(**B**) Western blots of nuclear/cytoplasmic fractions from HA-CARM1- (WT, S217E, S217A, S229E, or S229A) overexpressing cells. Anti-NFIBme2a antibody recognizes CARM1 substrates.

(**C**) Confocal images of CARM1 (red), CDK1 (green), and DAPI (blue) staining of 10T1/2 cells treated with RO-3306 or BI2536 for late G2 arrest.

(**D**) IP using anti-CARM1 antibody in cells transfected with HA-PKCα (WT, CA, or DN) for 48 h.

(**E**) IP using anti-CARM1 antibody in cells transfected with HA-CDK1 (WT or D146N) for 48 h.

(**F**) *In vitro* kinase assay using recombinant CDK1/Cyclin B1 protein and the CARM1(212-222 or 224-234) peptide.

(**G**) Western blots of cell lysates under the indicated conditions. After nocodazole pretreatment, calphostin C (0.5 µM, one hour), RO-3306 (10 µM, one hour), or BI2536 (100 nM, one hour) was added.

(**H**) IP using anti-GFP antibody in cells co-transfected with GFP-CARM1 and HA-CARM1 for 48 h and then treated with the indicated condition.

(**I**) Confocal images of p-CARM1 (S229) (red), CDK1 (green), and DAPI (blue) in 10T1/2 cells.


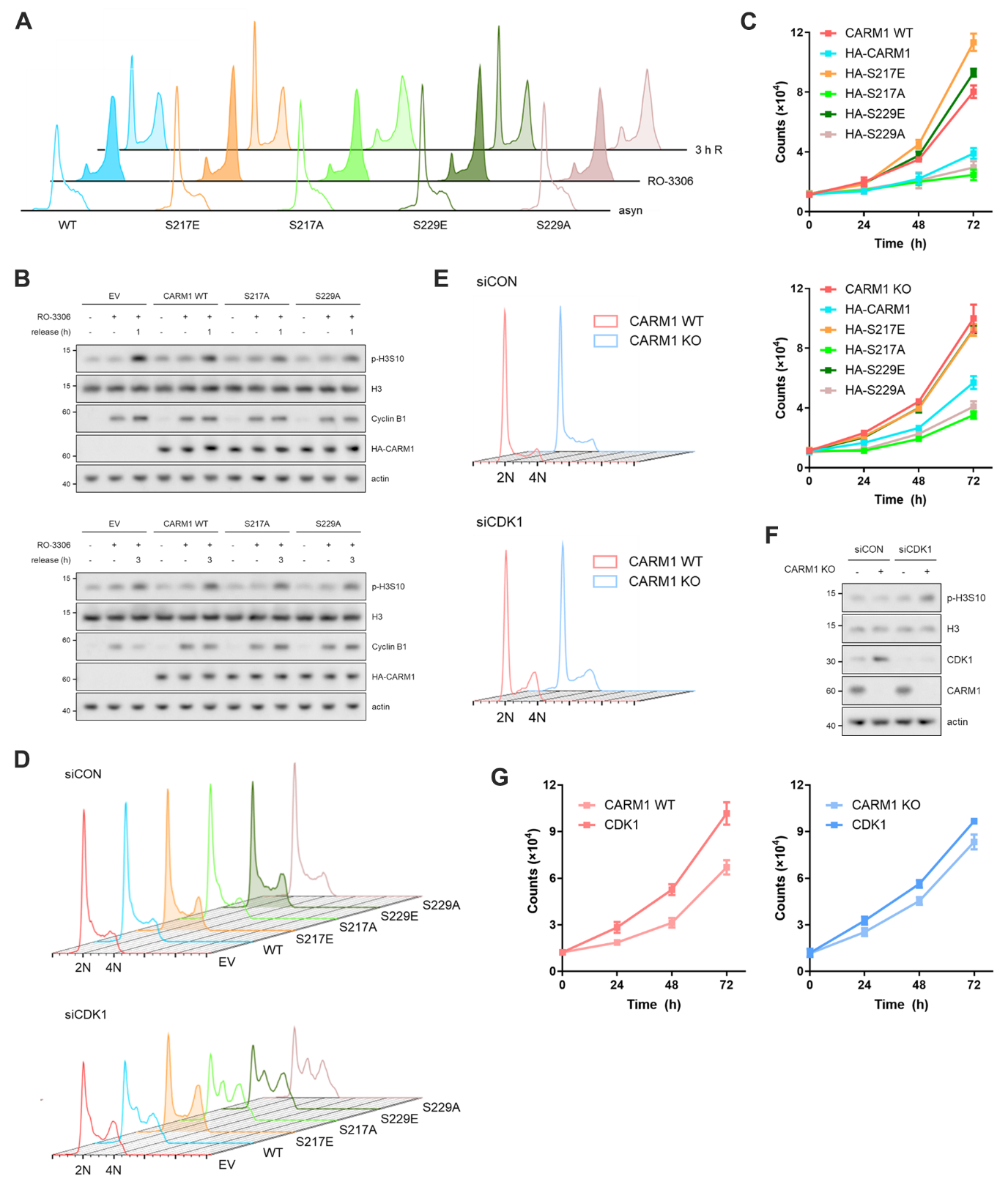


**Fig. S3. Mitotic progression requires an inactivation of CARM1 *via* S217 phosphorylation.**

(**A**) Cell cycle analysis using FACS from the release experiment after G2 phase arrest in HA-CARM1- (WT, S217E, S217A, S229E, or S229A) overexpressing cells.

(**B**) Western blots of cell lysates from the release experiment after G2 phase arrest in HA-CARM1- (WT, S217A, or S229A) overexpressing cells.

(**C**) Growth curves of CARM1-WT or -KO MEF cells transfected with HA-CARM1 (WT, S217E, S217A, S229E, or S229A) for 48 h. Error bars indicate SD of three independent experiments.

(**D**) Cell cycle analysis by FACS in cells co-transfected with HA-CARM1 (WT, S217E, S217A, S229E, or S229A) and CDK1 siRNA for 48 h.

(**E**) Cell cycle analysis using FACS in CDK1-depleted CARM1-WT or -KO MEF cells.

(**F**) The levels of p-H3S10 in CDK1-depleted CARM1-WT or -KO MEF cells were measured by western blotting.

(**G**) Growth curves of CARM1-WT or -KO MEF cells transfected with HA-CDK1 for 48 h. Error bars indicate SD of three independent experiments.


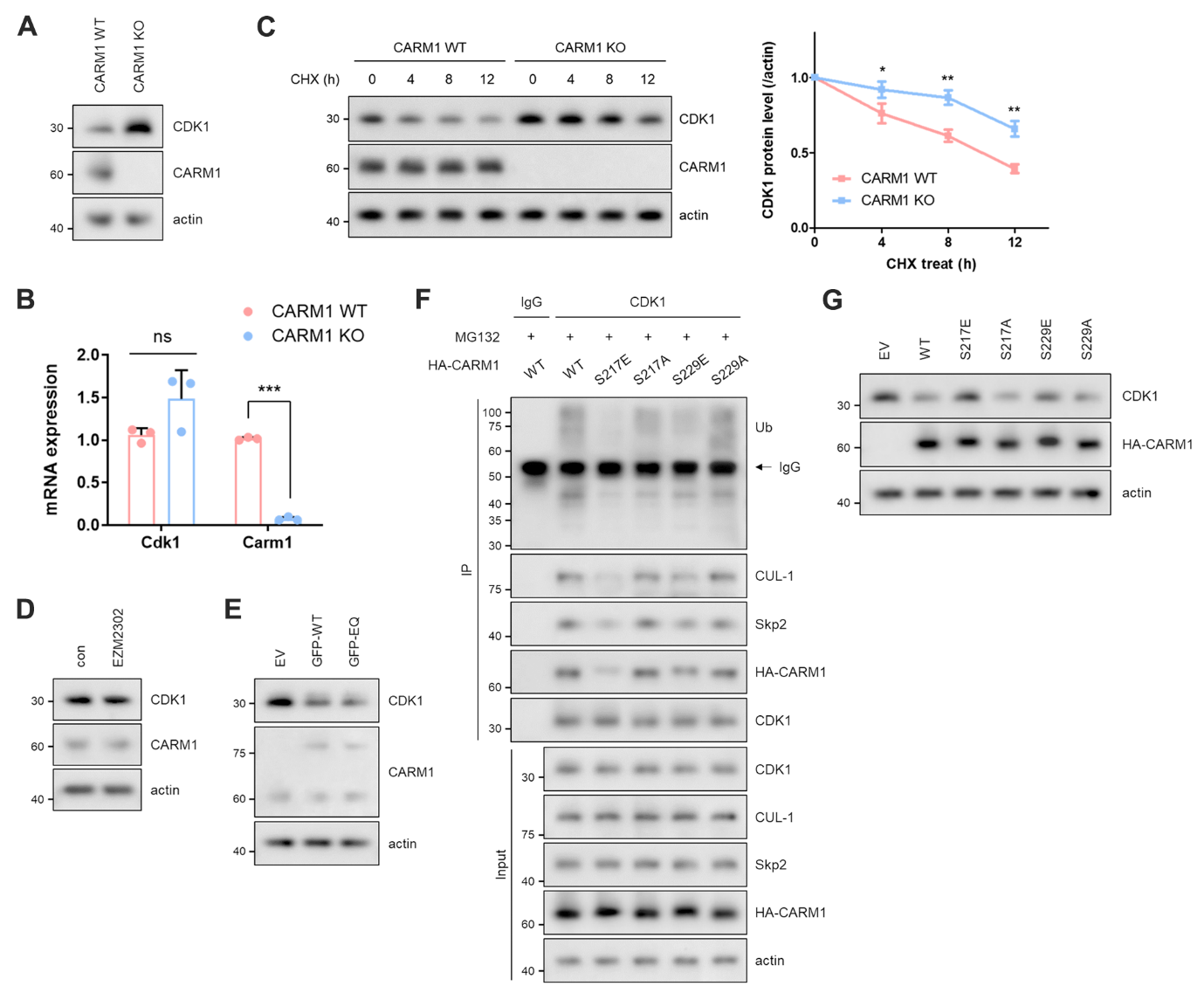


**Fig. S4. CARM1 functions as a scaffold for CUL-1-mediated CDK1 degradation.**

(**A** and **B**) Levels of CDK1 protein (A) and mRNA (B) in CARM1-WT or -KO MEF cells.

(**C**) Western blots of cell lysates from CARM1-WT or -KO MEF cells treated with cycloheximide (CHX, 50 μg/mL) for the indicated time. The error bars indicate the SD of three independent experiments.

(**D** and **E**) Western blots of cell lysates incubated with 1 μM EZM2302 for 72 h (D) or GFP-CARM1 (WT or E266Q) for 48 h (E).

(**F**) Western blots showing the interaction of CDK1 with CARM1/Skp2/CUL-1 complex and its ubiquitination in cells treated with MG132 (10 μM, six hours) after transfection with HA-CARM1 (WT, S217E, S217A, S229E, or S229A) for 48 h.

(**G**) Western blots of cell lysates from HA-CARM1 (WT, S217E, S217A, S229E, or S229A) overexpressing cells.
