# Supplementary figures and images for: CARM1 S217 phosphorylation by CDK1 in late G2 phase facilitates mitotic entry
Source: Cell Death Dis. 2025 Mar 25;16(1):202. doi: 10.1038/s41419-025-07533-z (PMC11937338; doi:10.1038/s41419-025-07533-z)

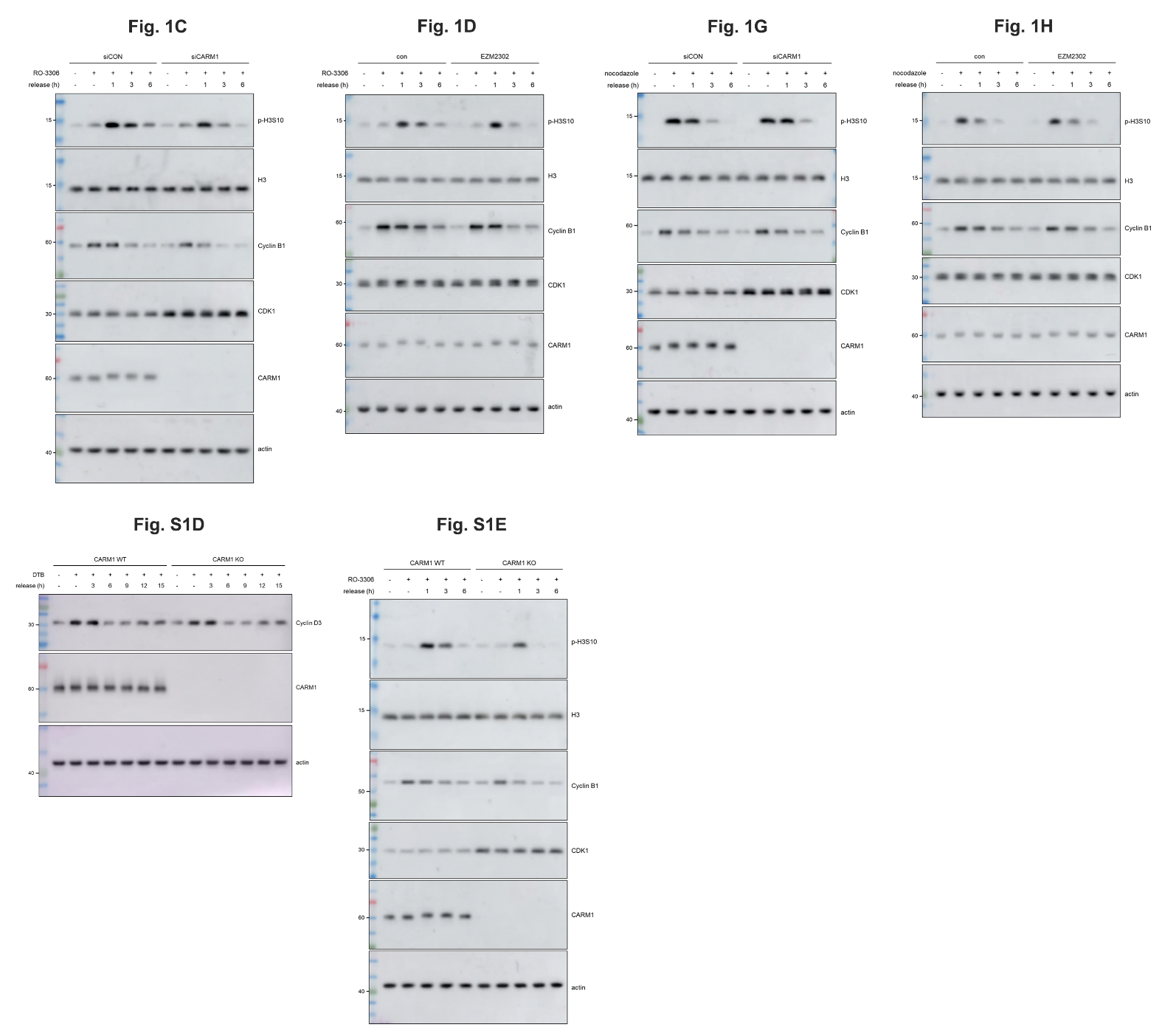

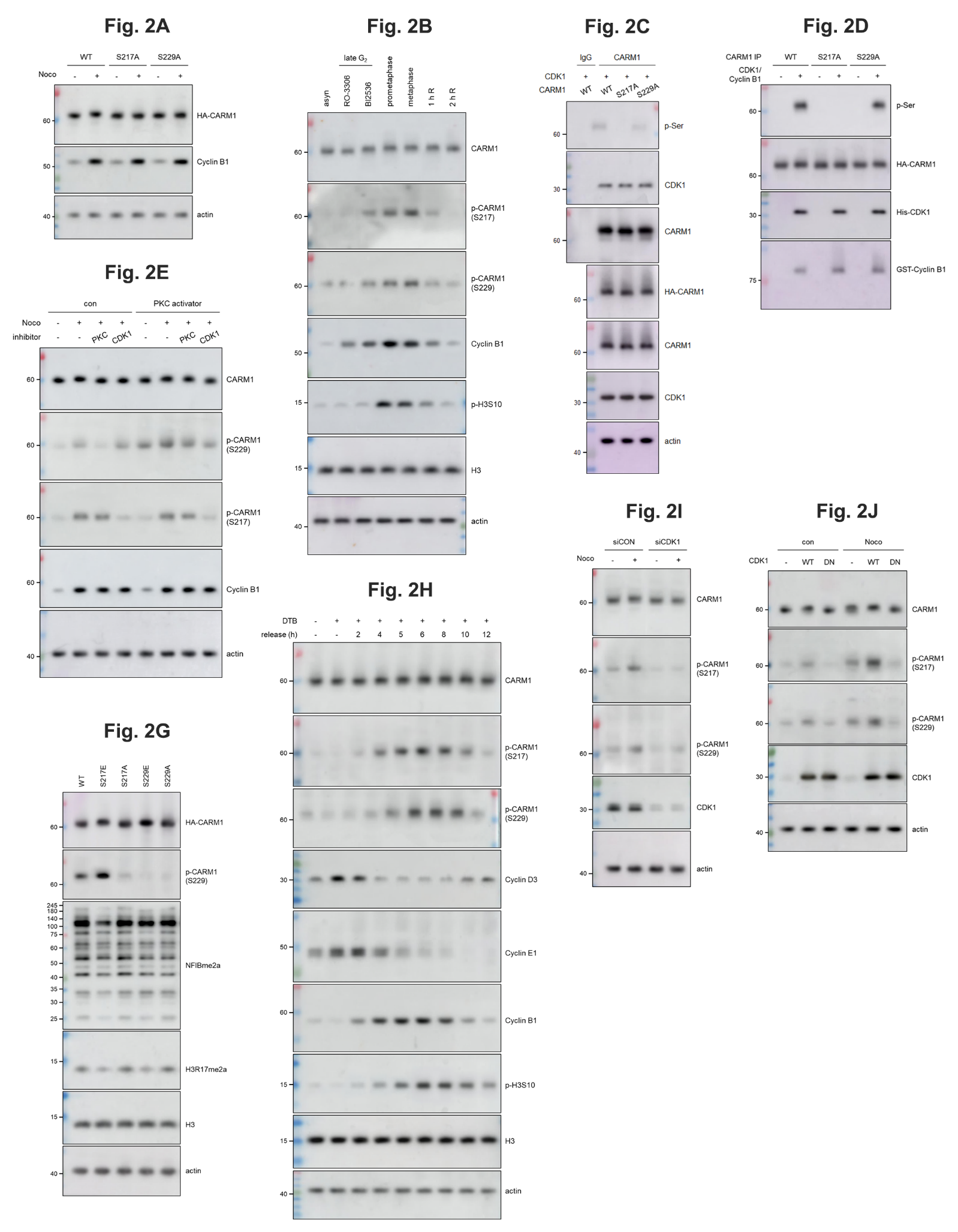

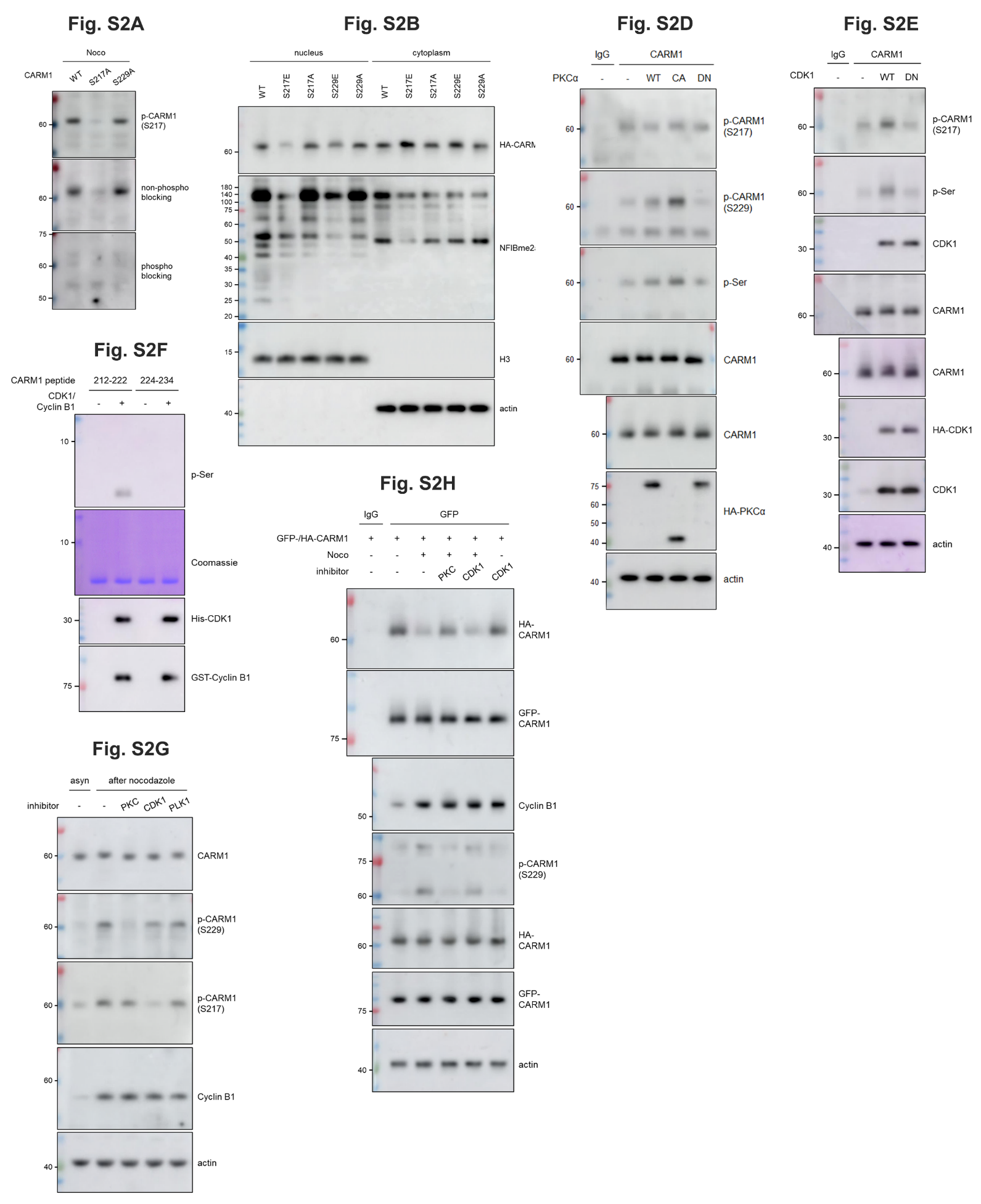

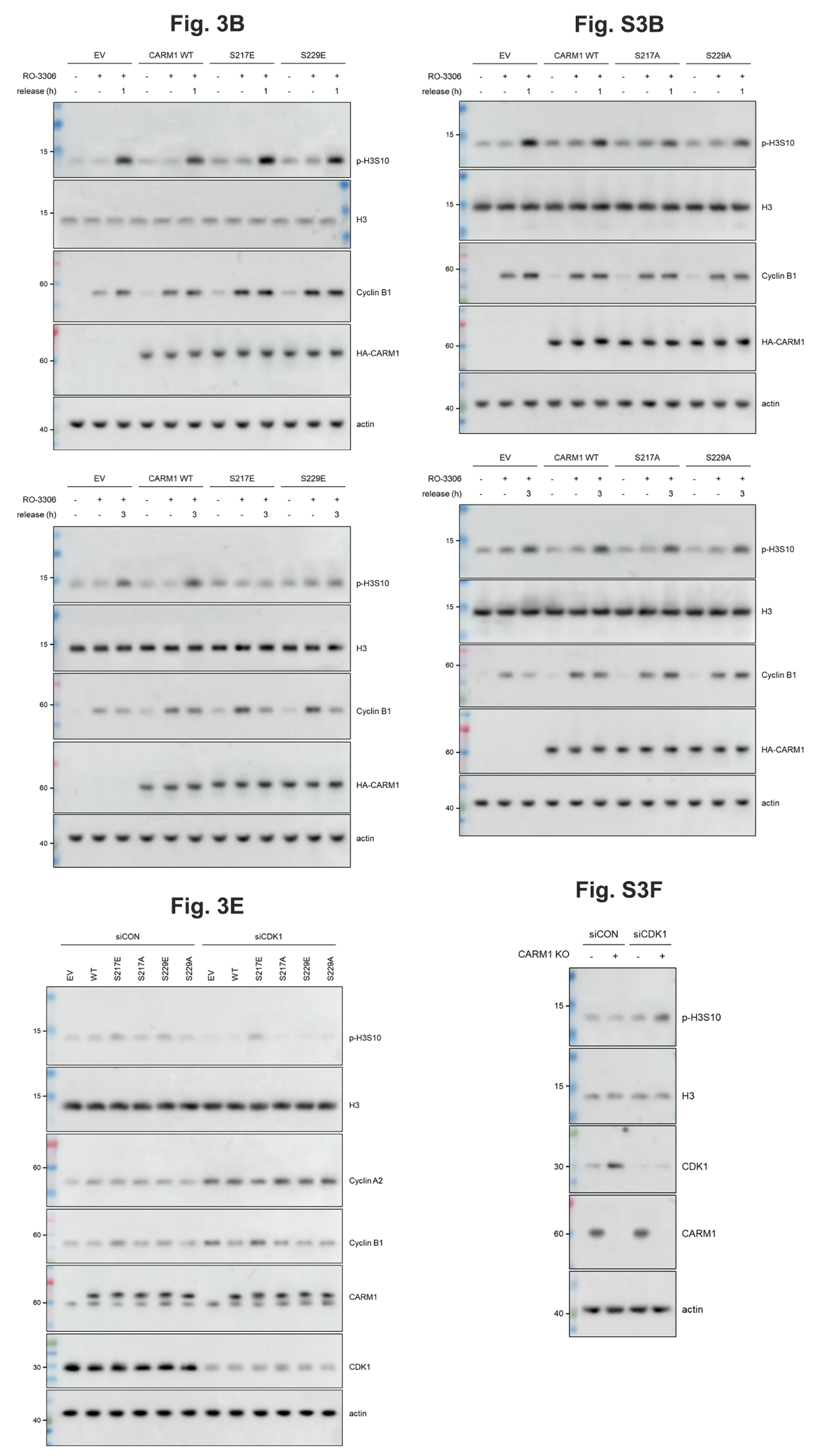

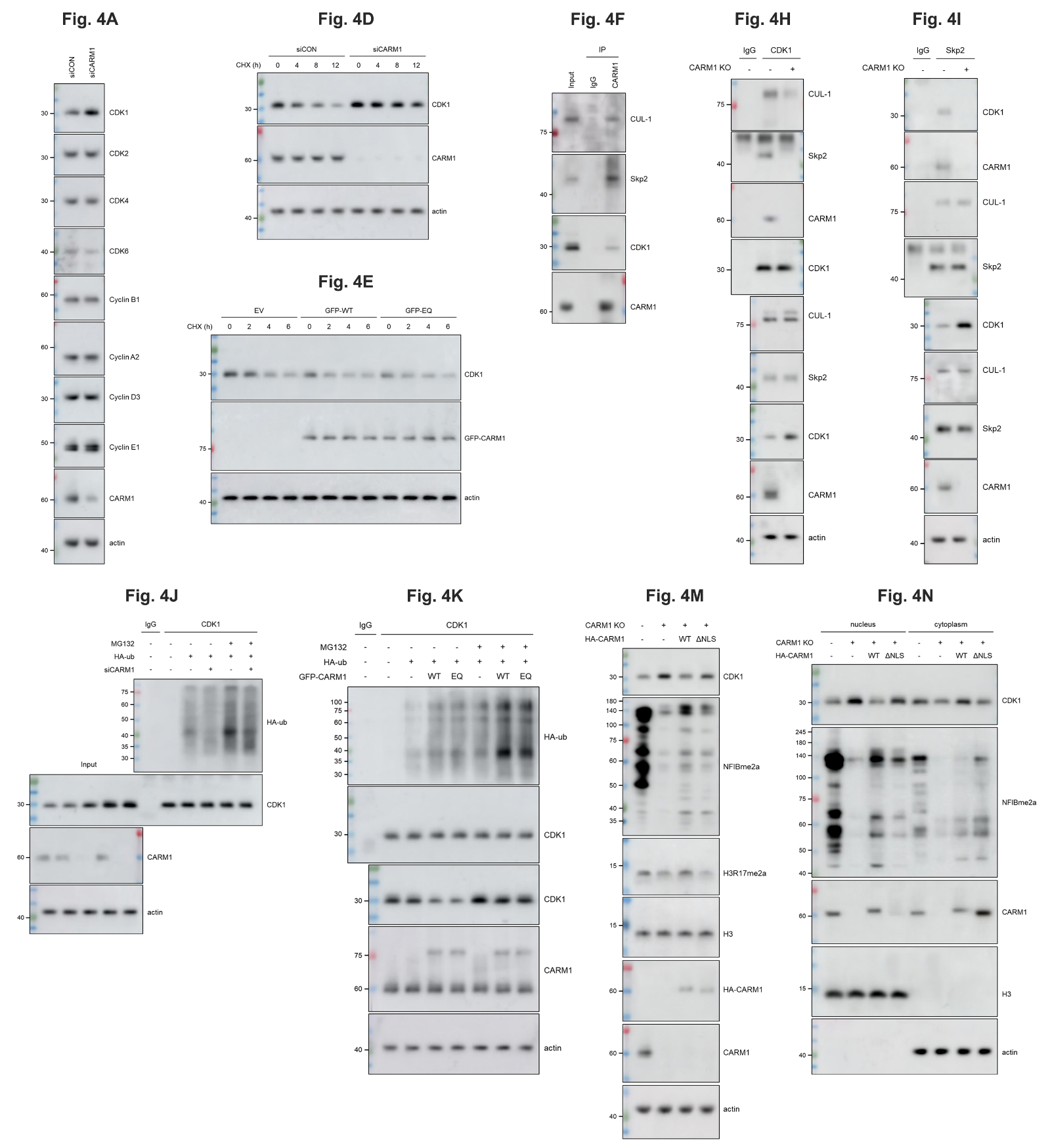

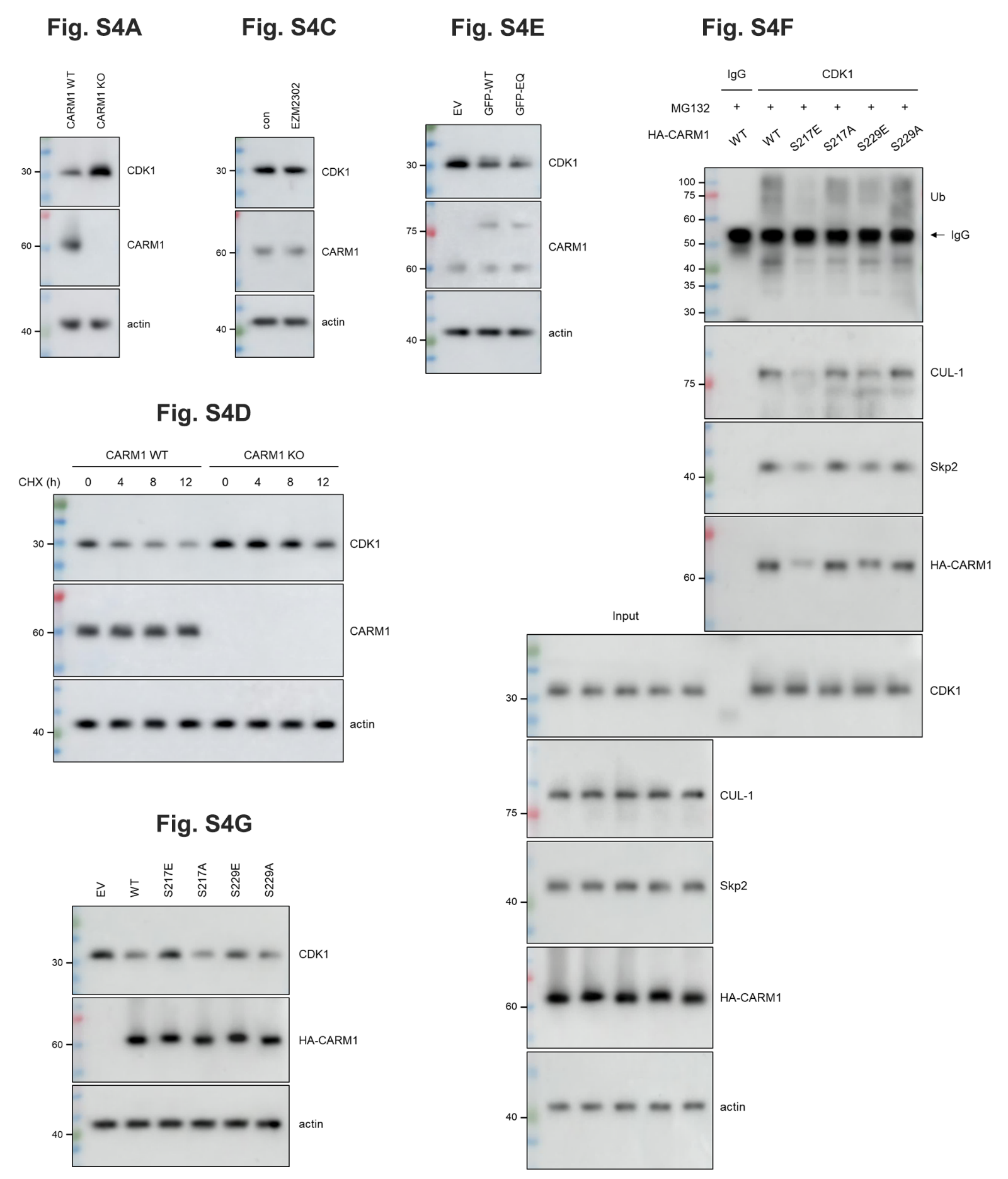

Supplement: Supplementary file 2 — Supplemental material - WB raw data [file 41419_2025_7533_MOESM2_ESM.docx]
